# Cardiac resynchronization performed by LBBaP‐CRT in patients with cardiac insufficiency and left bundle branch block

**DOI:** 10.1111/anec.12898

**Published:** 2021-09-22

**Authors:** Linna Zu, Zefeng Wang, Fei Hang, Yang Jiang, Xinlu Wang, Liting Cheng, Junmeng Zhang, Yongquan Wu

**Affiliations:** ^1^ Department of Cardiology Beijing Anzhen Hospital Capital Medical University Beijing China; ^2^ Department of Cardiology Aviation General Hospital China Medical University Beijing China; ^3^ Department of Cardiology Heart Center the First Hospital of Tsinghua University Beijing China

**Keywords:** cardiac insufficiency, cardiac resynchronization therapy, left bundle branch area pacing, left bundle branch block

## Abstract

**Objective:**

To evaluate the efficacy and safety of left bundle branch area pacing (LBBaP) in patients with heart failure and left bundle branch block (LBBB), and to compare the clinical effects with traditional cardiac resynchronization therapy (CRT).

**Methods:**

Thirty‐two patients with dilated cardiomyopathy complicated by cardiac insufficiency and left bundle branch block were divided into CRT group and LBBaP group. Parameters including pacing threshold, R‐wave amplitude, pacing impedance and operation time, and X‐ray exposure time were recorded. The left ventricular ejection fraction (LVEF), left ventricular end‐diastolic diameter (LVEDD), and left ventricular end‐systolic diameter (LVESD) were examined by echocardiography. The changes of QRS complex before and after operation were compared.

**Results:**

Compared with CRT group, the LBBaP group spent less time on total operation time and X‐ray exposure time and had stable electrode parameters including pacing threshold, R‐wave amplitude, and lead impedance after 12‐month follow‐up. In addition, LBBaP can achieve narrow QRS complex (117.15 ± 9.91) ms immediately than that in CRT group (130.32 ± 12.41) ms. The change of QRS between LBBaP is (50.30 ± 23.79) ms and CRT group is (33.15 ± 20.22) ms. After 6 months' follow‐up in LBBaP group, EF was higher than that before operation. Followed up for 12 months after operation, EF and LVEDD in LBBaP group were significantly improved compared with those before operation.

**Conclusion:**

Left bundle branch area pacing is a safe and effective resynchronization method for patients with cardiac insufficiency and asynchronization, which can achieve same clinical effects to CRT.

## 1 INTRODUCTION

Cardiac insufficiency is a serious manifestation of dilated cardiomyopathy, which affects the quality of life and life expectancy of patients. Although cardiac resynchronization therapy (CRT) is recommended by the guidelines as a recommendation for patients with left bundle branch block (LBBB) with cardiac insufficiency, the clinical 30% non‐response rate of CRT is a problem that cannot be ignored (Dickstein et al., 2008; Vijayaraman et al., 2017). Therefore, we were pursuing new effective treatment for dilated cardiomyopathy (DCM) patients with cardiac insufficiency all the time. In the year 2000, (Deshmukh et al., 2000) successfully performed His bundle pacing (HBP) on patients with atrial fibrillation accompanied by cardiac insufficiency but without intraventricular block with the help of steel wire and general active spiral electrode for the first time. This study was followed up for 2 years, and the results confirmed that the improvement of cardiac function in patients underwent HBP pacing was better than that of right ventricular pacing. Further studies have confirmed that 52% of bundle branch block (BBB) can be eliminated by HBP (Barba‐Pichardo et al., 2010); therefore, physiological pacing can effectively improve left and right ventricular electrical synchronization.

Physiological pacing is the best pacing mode we are pursuing at present, including His bundle pacing and left bundle branch area pacing (LBBaP). Because of the anatomical characteristics, the left bundle branch area is not enclosed by fibrous sheaths similar to those around the His bundle, the left bundle branch, and the Purkinje fibers are all exposed under the endocardium of left ventricle. Therefore, LBBaP has the advantages of lower threshold and more stable position over His bundle pacing (Chen et al., 2019; Zhang et al., 2019) and can correct left bundle branch block directly, so it is especially suitable for DCM patients with LBBB. With the development of assistive tools, implantation of electrode in left bundle branch area has become easier.

In this study, we retrospectively studied the improvement of cardiac electromechanical synchronization in DCM patients with cardiac insufficiency treated before and after left bundle branch area pacing, furthermore to explore the application prospects of LBBaP in the treatment of DCM patients with cardiac insufficiency.

## 2 PATIENTS AND METHODS

### 2.1 Patients selection

Thirty‐two patients with DCM complicated with cardiac insufficiency and LBBB from the Department of Cardiology, Beijing Anzhen Hospital, from March 2018 to May 2018, were enrolled. All patients were diagnosed DCM according to the European Dilated Cardiomyopathy Guidelines (Pinto et al., 2016) and have CRT indication (Ponikowski et al., 2016): QRS complex is more than 150 ms with LBBB, left ventricular ejection fraction (LVEF) <35% still has symptoms of persistent cardiac insufficiency symptoms after standard drug treatment, and ischemic cardiomyopathy was excluded by coronary angiography or coronary CTA within 1 year. In addition, the percentage of ventricular pacing was 98%–100% in all patients.

Patients with the following diseases were excluded: (1) bradycardia or malignant arrhythmia caused by reversible factors such as drug and electrolyte disorders; (2) acute myocardial infarction, acute cardiac insufficiency, severe liver and kidney insufficiency, acute and chronic infections, and other patients who are not suitable for surgery at present; (3) MRI findings in patients with myocardial fibrosis at the target electrode implantation site; (4) pregnant or lactating women; and (5) patients with mental disease or psychiatric disorder.

All patients were divided into CRT group and LBBaP group according to surgical methods. Implants in patients with left bundle pacing were performed by the same cardiologists. All the patients were informed of the operation method and signed the informed consent before operation. This study was approved by the Ethics Committee of Beijing Anzhen Hospital.

Before operation, basic information about the patients was collected, including gender, age, height, weight, past history including sick sinus syndrome (SSS), atrioventricular block (AVB), atrial fibrillation, coronary heart disease, hypertension, diabetes mellitus, cerebrovascular disease, and hyperlipidemia. QRS complex was measured, and echocardiographic data, including LVEF, left ventricular end‐diastolic diameter (LVEDD), and left ventricular end‐systolic diameter (LVESD), were collected. The pacemaker parameters including pacing threshold, R‐wave amplitude, and pacing impedance were observed after operation. The patients were followed up for 1 year. QRS complex and echocardiographic data were compared at 6 months and 12 months after operation.

### 2.2 Procedure

#### 2.2.1 LBBaP implantation

Left bundle branch area pacing was performed as described for the HBP method (Huang et al., 2017, 2019; Vijayaraman & Dandamudi, 2016). Briefly, with the aid of the C315/C314 sheath (Medtronic, Inc.), the selected Secure™ lead (model 3830; Medtronic, Inc.) was inserted into the His, and the His potential was measured. Images were obtained under X‐ray. Subsequently, the 3830 electrode and C315 sheath tube were pushed together in the apex direction (1–3 cm). When the 2 V output was unipolar paced by the 3830 electrode, the V_1_ QRS wave appeared W‐shaped, which was used as the electrode insertion point. Under the left anterior oblique position (LAO), the C315 sheath was adjusted in the vertical direction of the RV septum and the electrode was screwed into the chamber space under the X line. The electrodes were intermittently paced and the V_1_ QRS morphology (the W‐shaped "notch") gradually moved back until the vertical R wave appeared in the form of right bundle branch block (RBBB). In most cases, the intracardiac signals showed the Purkinje potential. The unipolar pacing was narrowed by QRS, showing a left anterior branch block pattern, which signified the successful implantation of the electrode.

**FIGURE 1:**
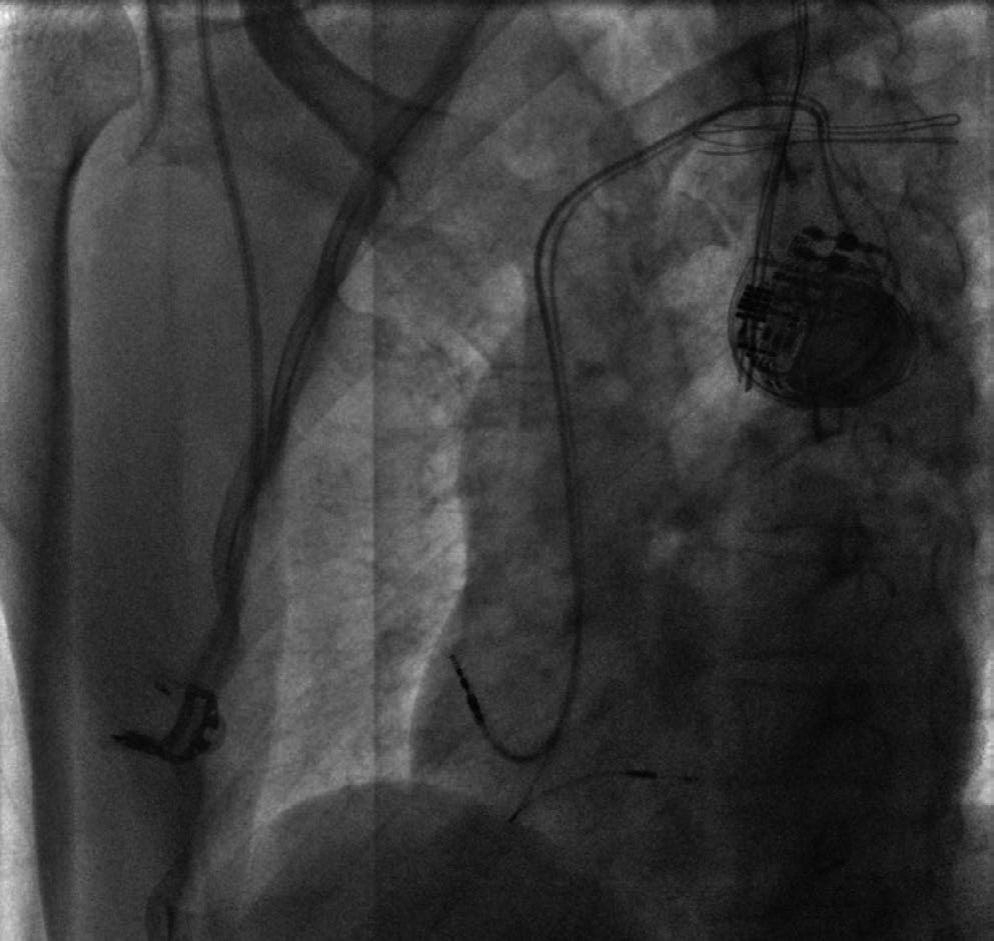
Pacing electrode of left bundle branch block was perpendicular to interventricular septum at left anterior oblique 40°

The basic criteria of LBBaP were as follows: (a) the duration of QRS <120 ms; (b) the pacing stimulus to QRS (S‐QRS) < native His‐QRS (H‐QRS); (c) the isoelectric line from stimulus to QRS onset was identified when pacing with a low output; (d) the duration of the stimulus to the ventricular activation peak (S‐Vmax) was similar for selective and non‐selective pacing; (e) the paced morphology was the left anterior branch block; and (f) after the successful implantation of LBBaP, the final position of the lower electrode was visible through X‐ray (Figure 1).

#### 2.2.2 CRT implantation

LV electrode was implanted into coronary vein by traditional way.

### 2.3 Statistics analyses

SPSS version 20.0 was used for all statistical analyses. Normally distributed continuous data were expressed as the mean ± SD. Categorical data were described as the number (%), and chi‐square test or Fisher's exact test (if the sample size was less than 40 or the minimum theoretical frequency was less than 1) and used to examine the aforementioned differences. All the tests were two‐sided. A *p*‐value <.05 was considered statistically significant.

## 3 RESULTS

### 3.1 Patients characteristics

A total of 32 consecutive patients were enrolled and divided into two groups according to the operation; finally, 19 patients underwent CRT. Three patients underwent LBBaP instead of previous CRT because of poorly ventilated target vein, so finally 13 patients underwent LBBaP.

The mean age of patients in the LBBaP group was (61.77 ± 12.37) years, and there were 8 (61.5%) males. The mean age of patients in the CRT group was (59.32 ± 5.41) years, and there were 15 (78.9%) males. There was no statistically significant difference in gender and age between the two groups. In addition, there were no statistically significant changes in comorbidities such as diabetes mellitus, hypertension, incidence of atrioventricular block, or electrocardiographic and electrocardiographic echocardiographic indices in the two groups. All the clinical baseline data did not statistically differ between the two groups (Table 1).

**TABLE 1:** Clinical baseline data

	LBBaP (*n* = 13)	CRT (*n* = 19)	*p*
Comorbidities			
Male (%)	8 (61.5)	15 (78.9)	.427
Age	61.77 ± 12.37	59.32 ± 5.41	.51
SSS (%)	1 (7.7)	0 (0)	.40
AVB (%)	4 (30.8)	2 (10.5)	.194
High blood pressure (%)	4 (30.8)	9 (47.4)	.471
Coronary heart disease (%)	1 (7.7)	6 (31.6)	.195
Hyperlipidemia (%)	2 (15.4)	2 (10.5)	1.00
Cerebrovascular disease (%)	1 (7.7)	0 (0)	.406
Atrial fibrillation (%)	1 (7.7)	5 (26.3)	.361
Diabetes mellitus (%)	2 (15.4)	5 (26.3)	.671
Electrocardiogram			
QRS complex	167.46 ± 28.11	163.47 ± 21.66	.654
Echocardiography			
EF	30.62 ± 6.983	29.11 ± 4.818	.474
LVEDD	66.23 ± 10.80	68.95 ± 12.37	.526
LVESD	55.69 ± 10.89	56.74 ± 13.68	.820

Note

Values are mean ± SD or *n* (%). *p* < .05 indicated statistically significant difference (Fisher's exact test).

Abbreviations: AVB, atrioventricular block; CRT, cardiac resynchronization therapy; EF, ejection fraction; LBBaP, Left bundle branch area pacing; LVEDD, left ventricular end‐diastolic diameter; LVESD, left ventricular end‐systolic diameter; SSS, sick sinus syndrome.

### 3.2 Operation time

The total operation time in LBBaP group (90.08 ± 33.40) min was significantly shorter than that of CRT group (158.05 ± 19.05) min, and the X‐ray exposure time in LBBaP group (20.46 ± 7.36) min was also significantly shorter than that in CRT group (43.53 ± 10.36) min (Table 2).

**TABLE 2:** Comparison of operation time between the two groups

	LBBaP (*n* = 13)	CRT (*n* = 19)	*p*
Total operation time	90.08 ± 33.40	158.05 ± 19.05	.00
X‐ray exposure time	20.46 ± 7.36	43.53 ± 10.362	.00

Note

Values are mean ± SD. *p* < .01 indicates statistically significant difference.

Abbreviations: CRT, cardiac resynchronization therapy; LBBaP, left bundle branch area pacing.

### 3.3 ECG characteristics

The QRS complex of patients in LBBaP group changed significantly. As LV electrodes rotated from right ventricle to left ventricular subendocardium through interventricular septum, the notch of lead V_1_ moved backward and upward gradually, and QRS complex changed from LBBB to RBB. LBB potential injury current was observed in 10 patients (76.9%).

QRS wave narrowed immediately after operation in both groups (Table 3). Average QRS complex of LBBaP group was (167.46 ± 28.11) ms before operation, and paced QRS complex was (117.15 ± 9.91) ms. The average QRS complex of CRT group is (163.47 ± 21.66) ms and paced QRS complex (130.32 ± 12.41) ms. The narrowing of QRS wave width confirmed a significant improvement in left and right ventricular asynchrony in both groups. The change of QRS in the LBBaP group (50.30 ± 23.79) ms vs. the CRT group (33.15 ± 20.22) ms was also statistically significant.

**TABLE 3:** Comparison of QRS (pre‐operation, post‐operation, QRS difference before and after operation) between the two groups

	LBBaP (*n* = 13)	CRT (*n* = 19)	*p*
Pre‐operation	167.46 ± 28.11	163.47 ± 21.66	.654
Post‐operation	117.15 ± 9.91	130.32 ± 12.41	.002
Difference before and after operation	50.30 ± 23.79	33.15 ± 20.22	.036

Note

Values are mean ± SD. *p* < .05 indicates statistically significant difference.

Abbreviations: CRT, cardiac resynchronization therapy; LBBaP, left bundle branch area pacing.

### 3.4 Echocardiogram characteristics

**FIGURE 2:**
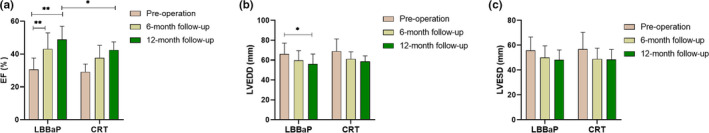
Comparison of postoperative echocardiogram (ECHO) characteristics between the two groups. (a) Ejection fraction (EF); (b) left ventricular end‐diastolic diameter (LVEDD); (c) left ventricular end‐systolic diameter (LVESD). *p < .05 and **p < .01

After 6‐month follow‐up, echocardiographic results showed that postoperative EF was significantly higher in the LBBaP group (43.15 ± 9.79) % than preoperative (30.62 ± 6.98) % (*p* < .01). In contrast, LVEDD and LVESD did not change significantly preoperatively and postoperatively (*p* > .05), and after 6‐month follow‐up, there was no significant difference in EF, LVEDD, and LVESD results between the LBBaP group and CRT group (Figure 2A‐C).

After 12‐month follow‐up, the postoperative EF (48.92 ± 8.06) % in LBBaP group was significantly higher than the preoperative one (30.62 ± 6.98) %, and LVEDD was also improved in the postoperative (56.00 ± 10.15) mm. LVESD did not change significantly in the preoperative and postoperative periods.

After 12‐month follow‐up, EF was significantly different between the LBBaP group (48.92 ± 8.06) % and CRT group (42.53 ± 4.89) %. However, LVEDD and LVESD were not significantly different between the two groups (*p* > .05) (Figure 2A‐C).

### 3.5 Pacing parameters

**FIGURE 3:**
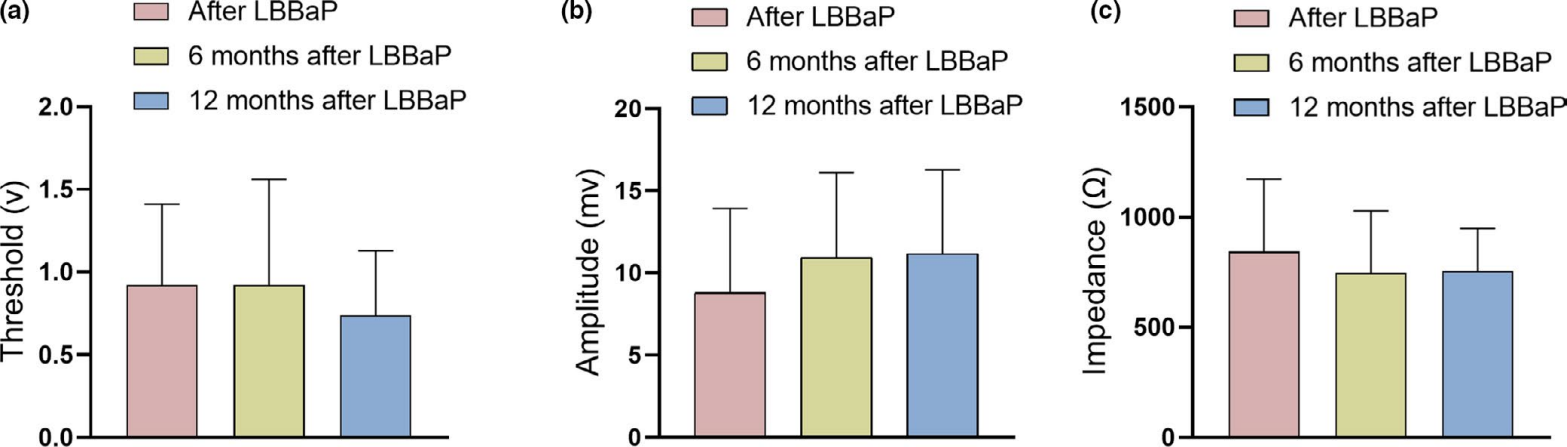
Comparison of pacing parameters in left bundle branch area pacing (LBBaP) group after operation, 6 months, and 12 months. (a) Pacing threshold; (b) R‐wave amplitude; (c) lead impedance

The pacing threshold, R‐wave amplitude, and lead impedance of left ventricular electrodes were stable after 6 and 12 months of follow‐up. The pacing threshold at postoperative, 6 months, and 12 months is (0.92 ± 0.49) mv, (0.92 ± 0.64) mv, and (0.74 ± 0.39) mv, respectively (Figure 3A). The R‐wave amplitude at postoperative, 6 months, and 12 months is (8.77 ± 5.15) v, (10.92 ± 5.18) v, and (11.15 ± 5.13) v, respectively (Figure 3B). The lead impedance at postoperative, 6 months, and 12 months is (842.62 ± 328.96) Ω, (745 ± 283.73) Ω, and (756 ± 191.52) Ω, respectively (Figure 3C). There was no significant difference between the three groups.

## 4 DISCUSSION

QRS wave is a direct and objective indicator of improvement, which can reflect the changes of cardiac electrical synchronization immediately. In this study, LBBaP was performed on patients with LBBB. QRS complex was significantly shorter immediately after operation. Preoperative QRS complex in LBBaP group was (167.46 ± 28.11) ms. After LBBaP was performed, we observed that QRS complex was significantly narrowed to (117.15 ± 9.91) ms. It can be seen that electrical synchronization can be achieved immediately after LBBaP. In addition, LBBaP group can achieve narrower QRS complex than that in CRT group that validated the previous research in our center (Zhang et al., 2019). We also compared the difference of QRS wave between the two groups and found that LBBaP group performed better. The change of QRS between LBBaP and CRT group also shows significant statistical difference, it can be seen that LBBaP group perform better on the improvement of QRS wave, and LBBaP achieves better effect on electrical resynchronization.

Cardiac resynchronization therapy is traditional method for the treatment of heart failure with biventricular asynchronization in which QRS wave width is greater than 150 ms. However, biventricular pacing is the fusion of left and right two‐point pacing, which is different from the normal conduction direction. The left ventricular electrode is located in the epicardium of the left ventricle, and cardiac excitation is from the epicardium to the endocardium, which is contrary to the physiological way of excitation from the endocardium to the epicardium. This is also the electrocardiographic basis of non‐response to CRT treatment. Physiological pacing has incomparable advantages compared with traditional pacing methods. In the 1970s, researchers attempted to stimulate His bundle in animal research and electrophysiological examination and succeeded in capturing His bundle. HBP is considered to be the most physiological mode of pacing. Compared with the traditional right ventricular apex pacing, HBP can significantly reduce the incidence of heart failure and rehospitalization rate in patients after pacemaker implantation (Abdelrahman et al., 2018; Ye et al., 2018). Research has shown that (Arnold et al., 2018) HBP can significantly shorten left ventricular activation time and improve myocardial electrical synchronization better than biventricular pacing, especially in patients with cardiac insufficiency associated with LBB.

However, there are some advantages of LBBaP over HBP. Huang et al., (2017) firstly performed LBBaP on a patient with heart failure and LBBB. They tried HBP during the operation at first, but LBBB could not be corrected. Later, the pacing site was moved forward 15 mm to the ventricle and paced again to correct LBBB. The pacing treatment was successfully carried out across the block area. The parameters including pacing threshold, R‐wave amplitude, and impedance were stable after the operation. Recently, Chen et al., (2018) confirmed that LBBP is a more physiological pacing mode than RVP. The pacing site is far from His bundle, which can surpass the blocking site, and the pacing range is relatively large. Therefore, the pacing threshold is low, the parameters are stable, and the safety is better.

In our study, we can see that the pacing threshold, R‐wave amplitude, and lead impedance of left ventricular electrodes were stable after 12 months of follow‐up. We carefully evaluated the electrode parameters at postoperative, 6 months, and 12 months after surgery; there was no significant difference among the three point of time. It can be seen that LBBaP is a safe pacing mode.

Professor Chen (Mafi‐Rad et al., 2016) also suggested that direct left ventricular middle septal pacing could be considered for LBBaP for those LBB could not be corrected or whose parameters were not good, that is placing the electrode at the middle part of the LV septum by transseptal approach which may achieve a relatively narrow QRS duration it can be an alternative way. In our study, LBBaP performed successfully, so this implantation method was not adopted. We evaluated the improvement of LBBaP on cardiac function by echocardiographic results. After 6 months' follow‐up in LBBaP group, EF was significantly higher than that before operation. Followed up for 12 months after operation, EF and LVEDD in LBBaP group were significantly improved compared with those before operation. And the results of EF and LVEDD between the two groups at 12 months' follow‐up were also significantly different. It can be seen that after 1 year of cardiac pacing treatment, LBBaP group performed better in the improvement of EF and LVEDD.

There is obvious advantages of LBBaP group over CRT group; LBBaP has shorter operation time and less radiation damage to the operator. In this study, it can be seen that the total operation time and X‐ray exposure time of LBBaP group are significantly shorter than those of CRT group. These findings concluded that LBBaP had better operability and was more friendly to the operators than CRT group.

Possible complications of LBBaP include pericardial effusion caused by perforation of free wall, tricuspid valve injury, acute myocardial infarction caused by injury of coronary artery, septal hematoma caused by injury of ventricular septal branch, and septal perforation caused by deep implantation. We closely observed the indicators above and evaluate the depth of electrode rotation during implantation, and there were no complications. So careful operation during the process can ensure the safe process of screw. So LBBaP is a relatively safe method as a new pacing mode. As LBBaP has a wide range of pacing site, and cross‐block site, 1‐year follow‐up shows that LBBaP has stable parameters, so we can see LBBaP is a promising surgical method which is worth widly spread, especially for those patients who cannot perform CRT or who cannot benefit from CRT.

There are some limitations in our study, Firstly, because LBBaP pacing is a relatively novel pacing mode, although some studies have confirmed that LBBaP is a feasible, safe, and stable pacing mode, large‐scale multi‐center and prospective studies are still needed to further evaluate it. Secondly, DCM patient accounts for the majority of CRT implantation patients. The small number of sample cases included in the study resulted in some results not being statistically different. Further larger clinical samples are needed for analysis. Finally, although the results confirm recent improvements in electrical and structural resynchronization, we still need longer follow‐up to evaluate parameters and long‐term structural remodeling after electrical resynchronization.

## 5 CONCLUSION

Left bundle branch area pacing group and CRT group can achieve the same effect in correcting left bundle branch block of ECG and improving cardiac function in patients with dilated cardiomyopathy. They can effectively shorten QRS wave duration and improve cardiac function. After a medium‐term follow‐up, LBBaP showed stable threshold and better improvement of QRS wave duration and improved cardiac function. Because of its shorter operation time and X‐ray exposure time, and simpler implantation process than CRT, it can be applied to patients with left ventricular electrode implantation difficulties and as a supplementary treatment for patients who cannot benefit from CRT.

## CONFLICT OF INTEREST

The authors claim that there is no conflict of interest.

## ETHICAL APPROVAL

This study was approved by the Ethics Committee of Beijing Anzhen Hospital.

## AUTHOR CONTRIBUTIONS

Linna Zu: Conceptualization, Methodology, Writing ‐ Review & Editing. Yongquan Wu: Writing ‐ Review & Editing. Junmeng Zhang: Methodolog, Formal analysis & Data Curation. Zefeng Wang, Fei Hang, Yang Jiang, Xinlu Wang, Liting Cheng: Formal analysis & Data Curation.

## Data Availability

The data that support the findings of this study are available from the corresponding author upon reasonable request.
